# Jump into a New Fold—A Homology Based Model for the ABCG2/BCRP Multidrug Transporter

**DOI:** 10.1371/journal.pone.0164426

**Published:** 2016-10-14

**Authors:** Laura László, Balázs Sarkadi, Tamás Hegedűs

**Affiliations:** 1 Molecular Biophysics Research Group and Department of Biophysics and Radiation Biology, Semmelweis University, Budapest, Hungary; 2 Research Centre for Natural Sciences, Institute of Enzymology, Hungarian Academy of Sciences, Budapest, Hungary; Russian Academy of Medical Sciences, RUSSIAN FEDERATION

## Abstract

ABCG2/BCRP is a membrane protein, involved in xenobiotic and endobiotic transport in key pharmacological barriers and drug metabolizing organs, in the protection of stem cells, and in multidrug resistance of cancer. Pharmacogenetic studies implicated the role of ABCG2 in response to widely used medicines and anticancer agents, as well as in gout. Its Q141K variant exhibits decreased functional expression thus increased drug accumulation and decreased urate secretion. Still, there has been no reliable molecular model available for this protein, as the published structures of other ABC transporters could not be properly fitted to the ABCG2 topology and experimental data. The recently published high resolution structure of a close homologue, the ABCG5-ABCG8 heterodimer, revealed a new ABC transporter fold, unique for ABCG proteins. Here we present a structural model of the ABCG2 homodimer based on this fold and detail the experimental results supporting this model. In order to describe the effect of mutations on structure and dynamics, and characterize substrate recognition and cholesterol regulation we performed molecular dynamics simulations using full length ABCG2 protein embedded in a membrane bilayer and *in silico* docking simulations. Our results show that in the Q141K variant the introduced positive charge diminishes the interaction between the nucleotide binding and transmembrane domains and the R482G variation alters the orientation of transmembrane helices. Moreover, the R482 position, which plays a role the substrate specificity of the transporter, is located in one of the substrate binding pockets identified by the *in silico* docking calculations. In summary, the ABCG2 model and *in silico* simulations presented here may have significant impact on understanding drug distribution and toxicity, as well as drug development against cancer chemotherapy resistance or gout.

## Introduction

ATP Binding Cassette (ABC) transporters form one of the largest families of membrane proteins and are involved in numerous physiological and pharmacological functions [1]. These proteins are present from bacteria to human, and while in bacteria they may work both as importers and exporters, in eukaryotes only the exporter function has been preserved. The human ABC protein family includes 48 members, from which the members of the ABCB, ABCC and ABCG proteins are key cellular exporters for xenobiotics and endobiotics [1, 2]. These promiscuous transporters provide the basis of protecting cells and tissue barriers against hydrophobic toxic materials, regulate the ADME-Tox (Absorption, Distribution, Metabolism, Excretion, and Toxicity) properties of numerous clinically applied drugs, and are involved in cancer chemotherapy resistance.

In spite of the huge amount of data about the mechanism of action, cellular localization, and pharmacogenetically important polymorphisms and mutations of the human ABC drug transporters, the structural features of these large membrane proteins are hardly known [3–5]. There are several crystallization-based experimental data for bacterial importers and exporters, while practically only one single set of such data are available for the mammalian (mouse) ABCB1 drug transporter [6]. Still, numerous homology models have been built for other human ABC drug transporters, either based on this ABCB1, or the bacterial ABC transporter structures [7–9].

The ABCG2 protein is a multifunctional human membrane transporter–it is expressed at a high level in the gut, in the blood-brain and the feto-maternal barriers, and its function is especially relevant in stem cell protection [10–12]. Overexpression of ABCG2 has been shown to cause cancer multidrug resistance [13–15], while a reduced expression or function is an established cause of hyperuricemia and gout [16–18]. Still, it has been especially challenging to model the atomic level structure of ABCG2, as the nucleotide binding domain (NBD) and the transmembrane domain (TMD) arrangements are in an inverse order than in the ABCB or ABCC families, and membrane topology studies indicated a completely different transmembrane helix arrangement for this protein.

Very recently the crystal structure of the ABCG5-ABCG8 heterodimer membrane protein, the key human transporter for cholesterol, has been published [19]. These proteins, as members of the human ABCG subfamily, show close homology to the homodimeric ABCG2 transporter. Since ABCG2 has major medical importance, here we provide a homology model for this protein, based on the ABCG5-ABCG8 structure. We also provide explanations for the polymorphism and mutations with experimental or clinical relevance [20–23]. Moreover, by utilizing molecular dynamics simulations and *in silico* docking calculations, we describe the potential drug binding and transport regions, as well as the residues responsible for the special cholesterol sensitivity of this promiscuous drug transporter. Clearly, these structural data may provide important clues to decipher the effects of the ABCG2 variants, design drugs to rescue their expression, or drugs to modulate their function in stem cell development, protection of the fetus or cancer drug resistance.

## Methods

### Homology modeling

Sequence alignment of ABCG2_HUMAN, ABCG5_HUMAN, and ABCG8_HUMAN (UniProt) was generated using ClustalW [24]. Modeller 9.12 was employed to generate the homology models [25]. Cysteines 592 and 608 were constrained to form intramolecular disulfide bonds, while cysteines at position 603 were forced to participate in an intermolecular S-S bridge. One hundred models were prepared and the best model was selected by Modeller’s DOPE score. Since the extracellular loops contained knots, the loops between C592 and C608 were refined also by Modeller, employing the same approach: 100 loop models were created and evaluated by DOPE score.

Mutant constructs could be generated simply by PyMOL (The PyMOL Molecular Graphics System, Version 1.7 Schrödinger, LLC.), since either the side chain of the new residue was small or it was on the protein surface. PyMOL was used to generate all the molecular graphics. Sequence alignments, structural models, and additional information on molecular dynamics and in silico docking (see below) can be downloaded from http://abcg.hegelab.org to facilitate further studies.

### Molecular Dynamics (MD) simulations

The structural models were oriented along the membrane normal based on the ABCG5-ABCG8 orientation in the OPM (Orientations of Proteins in Membranes) database [26]. MD simulations were performed using GROMACS 5.1 with CHARMM36 force field [27, 28]. The input files for energy minimization, several equilibration steps (NVT, NPT), and production run were generated via the CHARMM-GUI web interface [29, 30]. The following options were selected: terminal residues were patched by ACE (acetylated N-terminus) and CT3 (N-methylamide C-terminus), the extracellular cysteines were set to form disulfide bridges; homogenous POPC (1-palmitoyl-2-oleoyl-sn-glycero-3-phosphocholine) lipid bilayer were selected with default parameters and 150 mM NaCl was inserted; grid information for PME (Particle-Mesh Ewald) electrostatics was generated automatically, NPγT ensemble was selected with constant number of particles (N), pressure (P) of bar, surface tension zero (γ), and temperature of 310 K. All the wild type and mutant structures were energy minimized in the first step using the steepest descent integrator (maximum number to integrate: 5,000 or converged when force is <1,000 kJ/mol/nm). From each energy minimized structure we forked six parallel simulations containing consecutive sets of equilibration steps, when decreasing force constants (from 4,000 to 50 kJ/mol/nm^2^) in these steps were applied for protein and lipids. Production runs were performed without restraints. Berendsen thermostat and barostat were used in the equilibration steps, while Nose-Hoover thermostat and Parrinello-Rahman barostat with semiisotropic coupling were employed in the production run. Time constants were 1 ps and 5 ps for the thermostats and barostats, respectively. Electrostatic interactions were calculated using the fast smooth PME algorithm and LINCS algorithm was used to constrain bonds. Simulations were carried out in constant particle number, pressure, and temperature ensembles with a time step of 2 fs. In summary, all the parameters provided by the CHARMM-GUI interface were unchanged and used except the simulation time in the production step, that was set to 50 ns. Thus six parallel runs resulted in 300 ns total simulation time for each constructs including WT, Q141K, R486G, and Y413S. Simulations were executed on a GPU cluster of the NIIF National Information Infrastructure Development Institute (http://www.niif.hu/en). Simulations were analyzed by the MDAnalysis Python package [31] and in-house Python scripts on our local small HPC cluster.

### *In silico* docking

Molecules are listed in the Supplementary material (S6 Fig). The OpenBabel package and MGLTools scripts were used to convert between molecule file formats and to prepare the molecules for docking using AutoDock Vina [32, 33], respectively. The six conformations of ABCG2 wild type were taken from the last frame of the equilibrium simulations and prepared for docking using MGLTools (Gasteiger charges were added). The docking space was defined by a box around the whole transmembrane domain including also some parts of the NBD and the extracellular loops (S5 Fig). Because of the large volume of the box, exhaustiveness was set to 128 instead of the default value 8 and the number of required poses in the output (num_modes) was set to 20. Analysis was performed by PyMOL and in-house Python scripts.

## Results and Discussion

### Homology modeling based on the new ABC transporter fold provides a plausible and stable ABCG2 model

**Fig 1:**
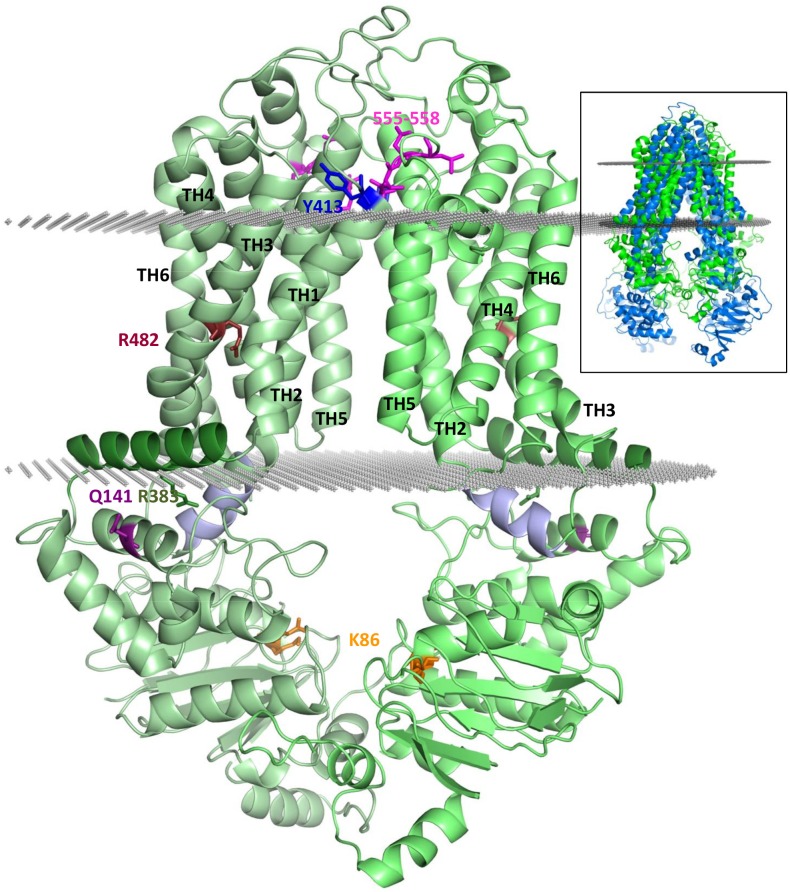
The general structural properties of the ABCG2 homology model. The two monomers are colored by different light green colors. The most important parts, providing the interface between the TMD and NBD are the coupling helix (light blue) and the connecting helix (dark green). The functionally important R482 is colored ruby. The site of the most frequent polymorphism, Q141 is deep purple. The location of important mutations affecting biogenesis and function are labeled by dark green (R383) and orange (K86), respectively. Residues, which are probably significant in cholesterol modulation, are blue (Y413) and magenta (a.a. 555–558). Gray dots represent the boundaries of the hydrophobic region of the bilayer, defined by the OPM webserver. **Insert:** ABCG2 (green) and mouse ABCB1/Pgp (blue, PDBID: 4M1M) are overlaid. The mouse ABCB1 NBD is much further from the membrane bilayer and the distance between the NBDs of ABCB1 and ABCG2 (the Cα atoms of the Walker A Lys residues; K433 and K86, respectively) is 26 Å.

In contrast to previous ABC transporter structures, the recent ABCG5-ABCG8 high resolution structure provides an excellent template for modeling ABCG2 [19]. ABCG2 exhibits 27% and 26% identities and 48% and 44% similarities when compared to ABCG5 and ABCG8, respectively. Although these values seem to be low for general homology modeling, for longer sequences (> 100 a.a.) and especially for membrane ABC proteins they are sufficient (see Modeller’s tutorial and [34]). Even in a worse scenario, when the N- and C-terminal halves of CFTR/ABCC7 (Cystic Fibrosis Transmembrane Conductance Regulator) exhibit only 18% and 21% identities compared to the *Staphylococcus aureus* Sav1866 protein, using this distantly related protein as a template resulted in a high quality CFTR homology model that could be confirmed by experiments [9]. Bacterial homolog based MDR1 (P-gp) models also have been generated and widely used to guide experimental and computational studies [35–37]. Although the ABCG5-ABCG8 template is a heterodimer, the structural differences between the two halves are subtle (S1 Fig). Based on these considerations, we generated a homology model of ABCG2 based on the ABCG5-ABCG8 structure (Fig 1) employing the sequence alignment shown in S2 Fig. Although the alignment generation was relatively straightforward, some parts of the ABCG2 protein were not modeled, either because they are mobile and thus invisible in the template structure, or their sequence and length differ from the corresponding regions in the template (e.g. the loop between the β1 and β2 strands of NBD and the linker region between the NBD and TMD; see details below and S2 Fig).

Large deviations from the starting structure are expected even in short (<20 ns) MD simulations when the initial structure is wrong or inaccurate [6, 38–40]. Therefore we performed molecular dynamics simulations employing the ABCG2 homodimer embedded in a membrane bilayer to confirm the stability of the homology model. RMSD values of frames compared to the initial structure indicated the stability of our model (S3 Fig). Although it would be interesting to perform experiments testing specific aspects of the ABCG2 structural model employing devised mutations, as in the case of the CFTR homology model [9], the ABCG2 transporter is highly sensitive to mutations and its cysteine-less form cannot be functionally expressed [41]. However, there are several experimental observations, which we discuss in the next section, supporting our model.

The nucleotide binding domains (NBDs) are the most conserved regions in all ABC proteins from bacteria to human, and consist of a RecA-like core domain present in all P-loop ATPases, and an α-subdomain characteristic exclusively for ABC proteins [4, 5, 42]. The Walker A and B motives responsible for ATP binding are located in the core domain, while the ABC signature sequence (LSGGQ), which provides the catalytic base toward the γ-phosphate, is situated in the α-subdomain (S2 Fig). Since ATP binds to the Walker A sequence in one NBD, and the signature sequence is provided from the other NBD, for ATP hydrolysis an intimate interaction of the two NBDs is required [4, 5, 42]. It is also important to mention that the ABCG5-ABCG8 heterodimer exhibits a functional asymmetry in the NBDs, as ABCG5 possesses a degenerate signature sequence thus unable to cleave ATP. In the ABCG2 dimer both ATP sites are active. The ABCG5-ABCG8 structure does not contain bound ATP, thus the NBDs were separated [19], and so are they now in the ABCG2 structure presented. However, in contrast to that in the mouse MDR1 structure [6], the NBDs are not fully separated, and a connection is provided by helices located at the C-terminus of NBDs. Till now this conformation could have been observed only in lower eukaryotes [43, 44].

In the ABCG type proteins the loop between the first and second β-strands of the core domain are longer than in most ABC transporters (approx. 20 a.a. in ABCG2, and 20 and 40 in ABCG5 and ABCG8, respectively; S2 Fig). This loop is invisible in both the ABCG5 and ABCG8 crystal structures, thus most likely highly mobile. The only loop, which has been described at the same location, is the so called “regulatory insertion” in the CFTR protein [45]. However, this region of the NBD does not play any role in phosphorylation or nucleotide dependent regulation of CFTR function. When this “regulatory insertion” was deleted in CFTR, the thermostability of this channel was increased significantly, but no physiological function for this segment could be identified [46]. Until now the existence of this long loop in ABCG proteins has not been known because of the lower sequence conservation of the β1-strand and its function is unknown. Since it includes the short A-loop motif [47], one of its functions supposed to be ATP stabilization. However, its length suggests additional roles, and we propose that it may serve as a filter at the entry to the substrate binding cavity (see below), and contribute to the first step of allosteric communication of signaling drug biding to the ATP binding site.

**Fig 2:**
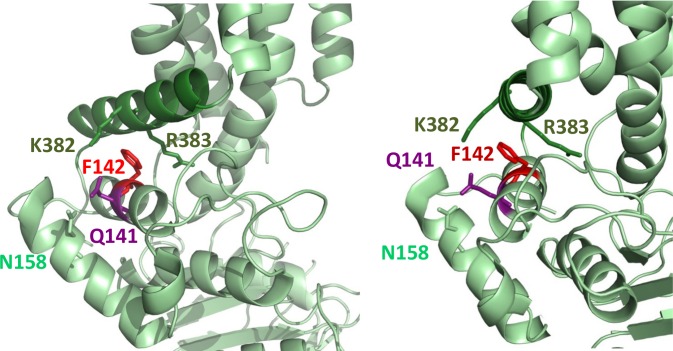
The Q141K variant interferes with the coupling between the NBD and the connecting helix. The side chain of F142, which is at a homologous position as the CFTR F508 (S2 Fig), is clamped by the positively charged K382 and R383. The positive charge of 141K destabilizes this interaction by repulsion with K382, as shown by molecular dynamics simulations (S5 Fig).

The transmembrane domain in the ABCG proteins exhibits a completely new ABC transporter fold. The intracellular loops are shorter compared to known ABC exporter structures, that results in a small distance of the NBDs from the inner layer of the membrane (Fig 1 insert). In this respect the structures of the ABCG proteins resemble the bacterial importers, but their transmembrane fold is completely different. In addition, no intracellular loops do cross over from one TMD to the opposite NBD, as observed in previous ABC exporter structures [9, 48]. Moreover, the arrangement of the short, so called coupling helices, which are located at the NBD/TMD interface and couple the motions resulting from ATP binding and hydrolysis in the NBDs to conformational changes in the TMD, are completely different from those found in the ABCB or ABCC type proteins. One of the intracellular loops between TH4 and TH5 (ICL2), in contrast to other ABC exporter folds, is so short that it does not leave the bilayer, thus does not reach the NBD and cannot function as a coupling helix, providing a molecular coupling. The only potential coupling helix in ICL1 (a.a. 452–461), located between TH2 and TH3, binds to the NBDs similarly to that observed in other ABC structures [9, 42] (Fig 1), but in a slightly different conformation. Interestingly, while there are bacterial importers that possess only one coupling helix in one half of the transporter, ABCGs still possess more interactions between the TMDs and NBDs. An amphipathic helix (a.a. 373–390; Figs 1 and 2 and S2 and S3 Figs) similar to a coupling helix provides additional interactions and is located in the linker region directly before TH1. The coupling helix like conformation of this region, which is named “connecting helix” [19] and forms an alpha helix perpendicular to the TM helices, is established by its amphipathic nature. However, this connecting helix does not penetrate as deeply into the NBDs as the coupling helices in Type I ABC exporters. In addition, this connecting helix exhibits similar structural and functional roles as those coupling helices which cross over from one TMD to the opposite NBD in the other half of the molecule, in type I ABC exporter structures.

An interesting and ABCG specific region of the new fold is an extracellular loop between TH5 and TH6 (ECL3; a.a. 552–621) with a special conformation. The residues located between 562 and 586 form two consecutive helices, exhibiting a V-shape, immersed partially into the bilayer (S2 and S4 Figs). This re-entering into the membrane resembles the P-loop of ion channels, and thus we propose to name it a G-loop. The extracellular loop following the G-loop in the ABCG proteins is not highly conserved and is slightly different in its length. ABCG8 does not possess any cysteines in this region, while the two cysteines in ABCG5 G-loop are at similar positions as C592 and C608 in the ABCG2. It has been experimentally documented that these cysteines form an intramolecular disulfide bond in the ABCG2 protein [49], while this covalent bond is not observable in the ABCG5-ABCG8 structure, because purification and crystallization were done under reducing conditions. In addition, in ABCG2 it has been shown that the two C603 residues in the two halves of ABCG2 form an intermolecular disulfide bond [49, 50]. The experimentally verified N-glycosylation site in ABCG2 at position N596 [51] in our ABCG2 model is found in the flexible extracellular loop 3 and accessible for glycosylation.

As described above, the ABCG family members have entirely different conformational arrangements in the transmembrane helices and connecting regions than the other known ABC exporters. Therefore it is not surprising that until now the structure of ABCG-like proteins could not have been properly predicted—all attempts were based on the presumption that the NBDs are located far from the bilayer and are connected to the TMD with long intracellular loops [8, 52–54]. In addition, experimental findings that supported the long ABC fold in ABCG type proteins may raise serious concerns (see Fig 1 insert). Moreover, studies which attempted to determine the ABCG2 transmembrane topology by HA-insertion experiments, seriously failed [54, 55]. This was most likely caused by a misinterpretation of the expression and function of the mutant variants, biased by the misconception of presuming long intracellular loops in TMDs. In contrast, the *in silico* prediction of the ABCG2 membrane topology performed much better, and resulted in TM helix boundaries similar to those deducted from the ABCG5-ABCG8 crystal structure and the present ABCG2 model (S1 Table) [56, 57].

### The new model contributes to the understanding of the effects of ABCG2 variations

Structural models, even homology models with their limitations, are important tools to assess the effects of both natural variations, and mutations generated for structure/function studies. A prominent example in the field is the CFTR homology model based on the Sav1866 structure [9]. While these proteins share very low homology in the TMDs, major structural features could have been confirmed by experiments and the model still serves as a fundamental basis from basic studies to drug development. Below we detail the experimentally examined specific residues and regions in the ABCG2 protein, in order to assess the suitability of the new model to understand their effects on ABCG2 structure and function.

The K86M mutation, within the Walker A ATP binding motif has been used in numerous studies to generate a non-functional ABCG2 transporter, lacking both ATPase and transport activities [58]. Similarly to other ABC transporters, the NBD/NBD domain interface is highly sensitive for mutations, irrespectively whether the actual residue plays a role in the catalysis. The E211Q mutation, causing loss of function, is localized in this region [59].

Two major polymorphic variants of ABCG2 are V12M and Q141K [22]. V12M, present in 5–10% frequency in human populations, has been shown not to have a measurable effect on the processing or function of the protein. The lack of effect can be explained by the position of this variation located in the short and flexible N-terminal region of the protein (a.a. 1–30). Tagging experiments are also in line with the spatial arrangement of the N-terminus: either a 6–10 histidine tag, or even a large GFP tag, attached to this end, is well tolerated in ABCG2 processing and function [60].

Q141K, present in about 15–30 percent of people in various ethnic groups, causes a significant reduction in intracellular trafficking and plasma membrane localization of ABCG2 [20, 21]. Due to the reduced expression at the site of action, this variant contributes to the development of gout and enhances the side effects and toxicity of various drugs [16–18, 61–63]. Interestingly, Q141 within the NBD is located next to F142, homologous to the CFTR F508 (Fig 2) [64], in a helix interacting with the amphipathic “connecting helix”. This site is analogous to the crossed-over coupling helix of cytoplasmic loop 3 in CFTR, and may similarly have a role in stabilizing the NBD/TMD interface [9, 65].

The side chain of Q141 is directed towards N158, thus the substitution of glutamine by the larger lysine with a positive charge, may displace the α-helix of N158. This helix is on the external side of the NBD, thus expected to cause only a minor effect on the transporter function. Molecular dynamics simulations indicate that these helices do not move differently in the Q141K mutant as compared to the wild type protein. On the other hand, the distances between the connecting helix and the helix of Q141 and F142 exhibit differences in dynamics (S5 Fig). The interaction at this NBD/TMD interface is stabilized by a special arrangement of three amino acids (Fig 2). The side chains of K382 and R383 in the connecting helix form a V-shape, and clamp the residue F142 located in the NBD. In the Q141K variant the positive charge interferes with K382, the interaction of the two helices is destabilized, and exhibits an increased probability of divergence for the two interfacing helices. In the light of this observation, the crucial role of R383 in stabilizing the NBD/TMD interface is highlighted and the deleterious impact of R383 mutations on ABCG2 biogenesis is interpretable [66].

Experimental studies have shown that while the Q141K variant causes only a partial impairment in ABCG2 processing, the ΔF142 mutant has a more severe effect than the ΔF508 mutation in the CFTR protein [64]. Misprocessing of ΔF142 ABCG2 cannot be rescued either by temperature or correctors, most likely because in the case of ABCG2 every single mutation behaves as a double mutation in the homodimer transporter. The molecular modeling and experimental studies may significantly contribute to explore the already available CFTR correctors to rescue the ABCG2 Q141K variant, e.g. in the therapy of gout [65]. Still, the local molecular environment is dissimilar to that observed in CFTR, namely the connecting helix is less embedded into the NBD, and the interacting residues are not hydrophobic in ABCG2, thus any CFTR corrector compounds tested for rescuing ABCG2 Q141K [65] should be tuned for the structure of ABCG2. In addition, CFTR F508 resides in a loop following a helix, while ABCG2 F142 is located in an α-helix.

In ABCG2 a historically and functionally important residue is the arginine in position 482 [58, 67]. In the first ABCG2 cloning experiments drug resistant cell lines expressed the R482G variant, which has a different substrate and inhibitor specificity and apparently a higher drug transport turnover that the wild-type protein. Still, this variant has not been found *in vivo*, probably because it cannot transport negatively charged substrates, including uric acid or conjugated hydrophobic drugs [23]. In the new ABCG2 model R482 resides in TH3, very close (2 a.a.) to a kink generated by P480. This proline, and most likely also the kink, are conserved in ABCGs (S2 Fig). As discussed below, the role of R482 in the substrate handling of ABCG2 is strongly supported by the current model.

The short cytoplasmic C terminus of the ABCG2 protein has been shown to be very sensitive to any experimental modification or tagging. This is well explained by the localization of these amino acids, facing the inner “cavity” of the dimer in very close proximity to the coupling helix and most probably interacting with it.

### Docking calculations reveal drug binding sites along a potential transport pathway

*In silico* docking studies on multidrug transporters have not been a great success. Since it is challenging to handle the plasticity of a binding site capable of interacting with chemically different compounds and *in silico* docking to a homology model raises the concern regarding side chain orientations, we performed docking to several conformations generated by the equilibration steps of the molecular dynamics simulations. This process also provided a more physiological orientation of the side chains in the lipid regions, as the protein in the simulations was embedded in a lipid bilayer. These conformations possess similar backbones, since the protein is position-restrained during the main part of the equilibration process. To these conformations we docked various ABCG2 substrates including sulfasalazine, methotrexate, rhodamine 123, flavopiridol, and also molecules that do not interact with ABCG2 such as verapamil and calcein (S6–S8 Figs) [2, 12, 15], employing AutoDock Vina [33].

**Fig 3:**
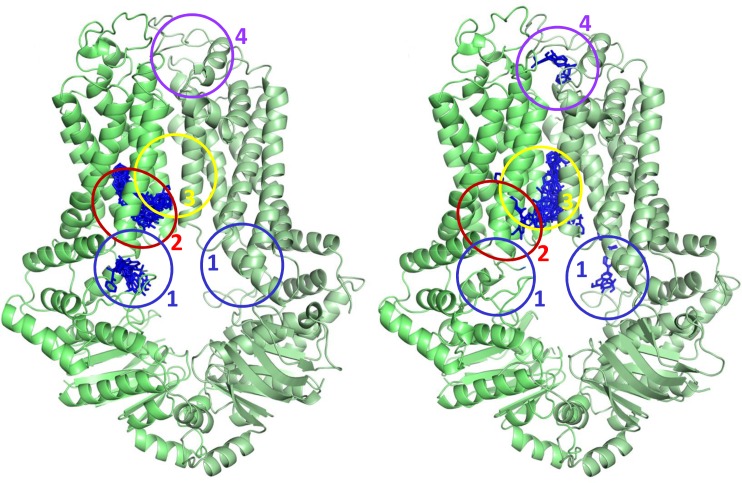
*In silico* docking shows binding sites along a potential substrate pathway. Substrates and non-substrates were docked to six ABCG2 conformations. Both types of molecules could dock at Site 1 (blue), while only the binding of substrates could be observed at Site 2 (red). The central Site 3 (yellow) resides between the two monomers. A potential off-site at the extracellular part is also revealed (Site 4, magenta). Here, docking poses of sulfasalazine are shown in the case of two ABCG2 conformations (two out of the last frames of the six equilibrations are shown).

Interestingly, the conformations exhibited various characteristic locations for substrates (Fig 3): some of the conformations indicated potential binding sites around the entry pore from the cytosol (Sites 1 and 2), others delineated the entry to the interface of the two transmembrane domains (Site 3), and some exhibited a partially extracellular location (Site 4). Site 1 is situated below the connector and coupling helix, and above the loop connecting the core and α-helical subdomains of NBD. Site 2 is a more defined binding pocket, intercalated between TH1 and TH4, which also includes the R482 and the P480 kink. Site 3 is located between the two monomeric subunits, surrounded by TH2 and TH5, provided by both monomers. Amino acids of Site 4 are part of the tip of the TH helices and extracellular loops. Binding to this site was a rare event, indicating that the conformations of this region captured in the MD simulations do not form a real binding pocket, as expected for an off-site.

Interestingly, while both substrates and non-substrates can bind to Site 1, the entry into Site 2 of non-substrates is limited (e.g. for verapamil or calcein; S7 and S8 Figs and S2 Table). Thus substrate selection, e.g. differentiation of toxic molecules from natural metabolites, may happen at this site. This proposal is also strengthened by the fact that R482, which exhibits a strong effect on substrate selectivity, is part of Site 2. It is also important to note that mutations of T402 and P485 in this pocket (S8 Fig) have been reported to reduce the transport of many substrates [8, 68], further supporting the existence of Site 2. It seems that all the molecules examined can bind into the central pocket (Site 3), which might be the entry point into the pathway between the two TMDs. In addition, for substrates, binding regions overlapping between Sites 2 and 3 also can be observed, delineating a potential transition spot from Site 2 to Site 3. Site 1 and Site 2 are present at both monomers, although their presence is not so pronounced in one of the monomers in our conformations because of the inherent asymmetry of the ABCG5-ABCG8 template. Site 3 is located between the two monomers, as a part of the main translocation path, and substrates can enter this pocket from Site 2 of either monomer in an alternating fashion (Fig 3). The central large cavity in the apo structures of ABCG5-ABCG8 and ABCG2 dimers exposes both hydrophobic and hydrophilic residues, and their pattern also may play a role in discriminating substrates and non-substrates. In addition, the loop between the β1 and β2 strands of NBDs may also limit the access to the entry sites and participate in substrate selection.

### *In silico* modeling facilitates to uncover the atomic details of cholesterol dependence of ABCG2 function

Membrane cholesterol is a major modulator of ABCG2 function, as documented by several experimental studies [69–71]. In fact, purified ABCG2 is practically non-functional without the addition of cholesterol to the reconstituting lipids [60]. Experimental studies indicated that the R482G mutation also influences the cholesterol sensitivity of ABCG2, that is less cholesterol is required for full transport function [69]. Since this amino acid is in the hydrophobic region, it is questionable how could cholesterol interact with this residue. Most probably R482 alters the conformation and dynamics of TM helices, resulting in altered cholesterol sensitivity and substrate specificity (see above).

**Fig 4:**
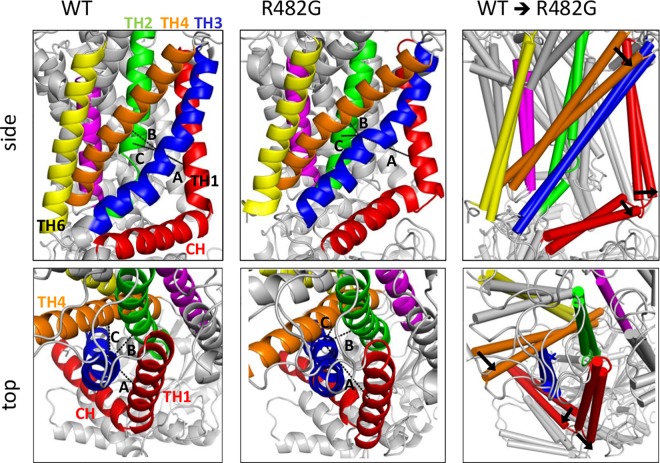
Structural effects of the R482 variations. The R482G mutation is able to alter the positioning of TM helices and the conformation of the P480 kink. Two structures were taken from the end of two simulations, which exhibited the largest changes, to decipher and demonstrate the effect of R482. The distances between Cα of R482 (TH3) and that of Q398 (TH1), S441 (TH2), and A517 (TH4) were measured throughout the simulation trajectory, and in the last frame exhibited the following values: distances of A, B, and C in WT are 8.4 Å, 7.2 Å, and 7.4 Å, while in the R482G variant are 15.1 Å, 8.1 Å, and 4.9 Å, respectively. The right panels contain both the WT and R482 structures in cylindrical representation. Arrows are placed at spots, which exhibit the most pronounced differences between the two constructs, and point from the wild type to the mutant conformation. TH1-6 are colored by red, green, blue, orange, magenta, and yellow, respectively.

In order to test this hypothesis we executed MD simulations using the R482G mutant embedded in a lipid bilayer, and compared the conformation of the TM helices close to this residue, located in TH3. Even in the case of a limited accessible time scale, large conformational changes could be observed (Fig 4 and S9 Fig). In the absence of the large R482 side chain, TH3 moved closer to TH4 and at the same time drifting away from TH1. These conformational changes caused by R482 substitution indeed have a significant effect on regions, which have been proposed as cholesterol binding sites (e.g. TH1), and also on drug binding site 2, thus provide suitable explanations for both altered cholesterol sensitivity and substrate specificity of this variant.

Two recent publications explored the putative cholesterol binding sites in ABCG2 by mutagenesis. Gal *et al*. [72] investigated the effects of mutations of the key tyrosine residues in the putative cholesterol recognition amino acid consensus (CRAC) motives, located in ABCG2 at positions Y413, Y459, Y469, Y570 and Y645 (S4 Fig). The Y459S mutation prevented protein expression, the Y469S and Y645S mutants lost their transport and ATPase activities, while the only significant effect on cholesterol modulation of ABCG2 function was caused by the Y413S mutation. In the second related study [73] a leucine based potential cholesterol binding motif (a.a. 555–558) was found to play a significant role in the cholesterol dependence of ABCG2.

**Fig 5:**
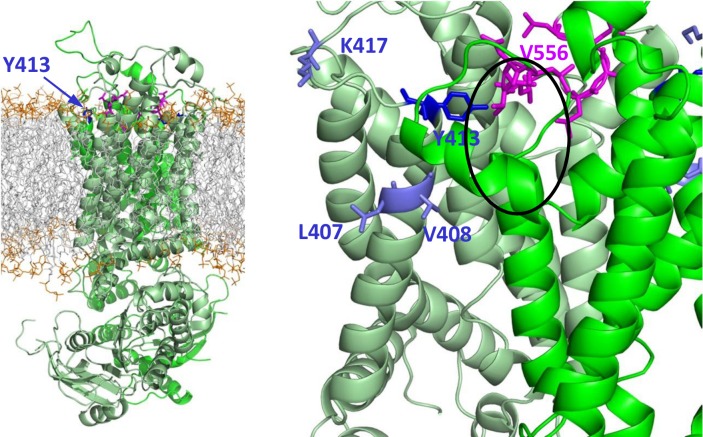
The structural background of cholesterol regulation. The last frame of a 50 ns long MD simulation with ABCG2 embedded in a POPC bilayer shows that the CRAC motif, containing Y413, is located in the charged area of POPC head groups (orange), as a rational location for cholesterol biding. Also, the leucine based cholesterol binding motif (magenta) is situated in this layer. Right panel: zoomed area reveals a close contact between the CRAC and the leucine based motives (e.g. Y413 and V556 are closer than 5 Å) and may provide a cholesterol binding site (black circle). Gray: POPC hydrophobic tails; orange: charged head groups of lipids.

The structural model of ABCG2 presented here revealed that Y459 is located in the coupling helix and its mutation understandably caused a major detrimental effect. The Y413S CRAC motif mutation, that significantly altered the cholesterol sensitivity of ABCG2 without major functional effects, is located in the extracellular tip of TH1, in the area of the external lipid head groups, as supported by molecular dynamics simulations, performed with the transporter embedded in a POPC lipid bilayer (Fig 5). Most interestingly, the leucine based motif (a.a. 555–558), affecting the cholesterol sensitivity of ABCG2 is located just before the G-loop, in the outer, charged leaflet of the bilayer, close and in a potentially interacting position with the Y413 in the CRAC motif in the opposite monomer. These observations strongly suggest that the cholesterol binding site is located in this region. It may be supposed that an intermolecular interaction between Y413, L555, and V556 provide a specific, potentially dynamic conformation for the TM helices in cholesterol binding. The special localization of these two motives also suggests that cholesterol may contribute to the stabilization of the dimer in a specific conformation.

Since the Y413S mutation may affect cholesterol sensitivity of ABCG2 allosterically, and experimental studies to identify the direct binding of such a hydrophobic molecule are extremely difficult, we performed MD simulations with the Y413S mutant construct. We could not observe any significant changes in the dynamics in TH1 (S10 Fig), that also suggests that the altered cholesterol sensitivity observed in this mutant is caused directly by this functional CRAC motif.

## Concluding remarks

The ABCG5-ABCG8 based homology model of ABCG2 presented here is capable to shed light on the effects of mutations, substrate handling, and also on the regulation of this transporter. The altered dynamics of the R482G variant provides explanation for both altered substrate specificity *via* affecting the drug binding pocket (Site 2), and to the altered cholesterol regulation through allosteric communication via TH1 to CRAC motif (Y413). As this ABCG5-ABCG8 based homology model behaved unexpectedly well in the simple computational approaches we applied, without any sophisticated additional methods, most likely the ABCG5-ABCG8 structure is a physiologically relevant conformation. Importantly, this conformation exhibits only slightly separated NBDs without bound ATP and exposes drug binding sites without widely separated TM helices at the cytoplasmic membrane leaflet.

The existence of the observed binding sites in the six conformations used in *in silico* docking calculations leads to important implications. Namely, subtle conformational changes (the maximum RMSD between the six equilibrated structures is 1.3 Å) are sufficient to provide binding sites at different regions of the protein, without the need of large movements or largely separated NBDs. Most likely the alternating access mechanism, which has been proposed to involve a drug binding conformation with widely separated intracellular parts and a release conformation widely opened to the extracellular space, is realized differently in the case of ABCG exporters. Based on the ABCG5-ABCG8 structure and our results with the ABCG2 model we hypothesize that the conformational changes needed for transport involve subtle repositioning and rotation of the transmembrane helices and these play a more pronounced role in the transport than have thought before. Supported by our results we are convinced that the ABCG2 model presented here may pave the road for drug design and understanding multidrug recognition and transport.

## Supporting Information

S1 Fig Structural alignment of ABCG5 and ABCG8. Chain A (ABCG5, green) and chain B (ABCG8, blue) from the heterodimer structure (PDBID: 5DO7) are highly similar (RMSD 2.1 Å) except in the linker region.
(TIF)

S2 Fig Sequence alignments of ABCG subfamily members and CFTR NBD1. **(A)** Sequences of the ABCG proteins downloaded from the UniProt database (ABCG1_HUMAN, ABCG2_HUMAN, ABCG4_HUMAN, ABCG5_HUMAN, and ABCG8_HUMAN) were aligned employing ClustalW. ABCG2 N-terminus including NBD (a.a. 1–300) and CFTR NBD1 (CFTR_HUMAN, a.a. 381–645) were aligned separately, since CFTR NBD1 sequence has unique features, which result in suboptimal alignments even for demonstration purposes when aligned together with all ABCG proteins. The CFTR NBD1 was merged manually from this pairwise alignment into the multiple alignment. Since the CFTR transmembrane domain is a Type I exporter fold and not similar in either length or sequence to ABCG proteins, this TMD is not included in the alignment. Important regions and amino acids positions are labeled above the alignment and their numbering refers to ABCG2 positions. Since ABCG2 exhibit somewhat higher similarity to ABCG1 and ABCG4, the alignment of ABCG2 to ABCG5 and ABCG8 is not optimal at a few minor positions (labeled with red arrowheads). **(B)** We also generated pairwise alignments of the ABCG2 to the templates that was used for homology modeling and contains concatenated dimers of ABCG2 (labeled as ABCG22) and ABCG5-ABCG8 (labeled as ABCG58). The loop between the NBD β1 and β2 strands are highly different in length resulting in highly misaligned sequences of this region, thus the β1 sequence of ABCG8 was manually aligned to the β1 sequence of ABCG2. The linker region was not modeled because of low sequence similarities and its flexible missing parts in ABCG5-ABCG8. The ABCG2 sequence exhibits sufficiently high similarity to the templates (identity ~25% and similarity over 40%) that allows generating high quality homology models in the case of these ABC transmembrane proteins (Modeller tutorial and [34]). The alignment was drawn using Jalview, colored according to the ClustalX color scheme, and can be downloaded from http://abcg.hegelab.org. The monomer’s boundaries are indicated by red arrowheads, while locations of breaks in the structure are indicated by black arrowheads.
(TIF)

S3 Fig MD simulations show the stability of the homology model and the mutant constructs. We performed six parallel 50 ns long MD simulations with every constructs embedded in a lipid bilayer. All of them exhibited a stable structure, with sufficiently stabilized RMSD and energy values that can be considered acceptable for such a large and stable system. Major distortions, which were published for MD simulations with crystal structures of other ABC transporters, could not be observed in the case of our structural model [38,39].
(TIFF)

S4 Fig Important regions and amino acid locations are highlighted on the ABCG2 model. Cysteines (red) were constrained to form intramolecular (C592-C608) or intermolecular (C603-C603) disulfide bonds. The glycosylation site N596 (black) resides in a mobile loop indicated by molecular dynamics simulations. Amino acids between 562 and 586 form two consecutive helices, which are immersed partially into the bilayer and resembles to the P-loop of ion channels, thus we propose to name it as a G-loop (yellow). CRAC motives are labeled blue and the coupling helix light blue. The tyrosines in the potential CRAC motifs (Y413, Y459, Y469, Y570 and Y645, see text) are colored blue. R482 and P480, which proline creates a kink in the helix, are colored ruby and pale green, respectively.
(TIFF)

S5 Fig The Q141K mutation may cause repulsion with K382 and weaken the NBD/TMD interface. Although the Q141 side chain points towards N158 (Fig 2), the Q141K mutations does not exhibit a pronounced effect of the distances of the helices, in which these amino acids are located (not shown). However, the positively charged 141K interferes with the side chain of K382, which restrains F142 with R383 (see Fig 2). This is revealed by MD simulations showing increased distances and higher variability of distances between the Cα atoms of restudies 141 and 382 in the Q141K variant, as compared to the wild type protein. All the 5,000 frames were analyzed and every consecutive ten distance values were averaged to smooth the plots of all distance measurements in this study.
(TIFF)

S6 Fig Compounds selected for *in silico* docking. 3D and 2D structures were downloaded from PubChem and ChemSpider. ABCG2 **s**ubstrates: Acetaminophen-sulfate (CID: 83939), Acyclovir (CID: 2022), Afatinib (CID: 10184653), Albendazole-sulphoxide (CID: 83969), Arry-334543 (CID: 42642648), Benzo(a)pyrene (CID: 2336), Benzoylphenylurea (CID: 74566), Ciprofloxacin (CID: 2764), D-Luciferin (CID: 5484207), Danusertib (CID: 11442891), Eltrombopag (CID: 9846180), Flavopiridol (CID: 5287969), Icotinib (CID: 22024915), Masitinib (CID: 10074640), Methotrexate (CID: 126941), N-Acetyl-amonafide (CID: 10064887), Pheophorbide A (CID: 5323510), Protoporphyrin IX (CID: 4971), Purpurin18 (CID: 5489047), Rhodamine123 (CID: 65218), Rosuvastatin (CID: 446157), Sulfasalazine (CID: 5359476), Tandutinib (CID: 3038522), Telatinib (CID: 9808844), and Uric-Acid (CID: 1175). Non-substrates: Calcein (CID: 65079), Calcein AM (ChemSpiderID: 346571), Colchicine (CID: 6167), Digoxin (ChemSpiderID: 206532), Doxycycline (CID: 54671203), Fluo-3 (ChemSpiderID: 94730), LTC4 (ChemSpiderID: 4444133), NEM-GS (CID: 443150), Ketoconazole (CID: 456201), Loperamide (CID: 3955), Quinidine (CID: 441074), Reserpine (CID: 5770), Verapamil (CID: 2520), and Vinblastine (ChemSpiderID: 12773).
(TIF)

S7 Fig Non-substrates are limited in accessing binding Site 2. We performed *in silico* docking calculations for flavopiridol and methotrexate, two established ABCG2 substrates (green labels) as well as verapamil and calcein, which are not transported substrates of ABCG2 (red labels), to the six equilibrated conformation of the transporter. Although we could observe binding to Site 1 (blue) for all compounds, access of verapamil to Site 2 (red), which is the binding pocket including R482, was decreased compared to that of substrates. Calcein also exhibited a limited access to this site, without deep penetration into it. These observations suggest that these ABCG2 conformations can be employed in future studies for developing *in silico* methods to distinguish substrates and non-substrates. Moreover, Site 2 may be the gate that differentiates between toxic molecules and tolerated metabolites. The black box indicates the search space defined in all of our docking calculations. Docking to one of the six equilibrated conformations is shown.
(TIFF)

S8 Fig Quantitative analysis of sites of a potential translocation pathway. After visual inspection of all the binding poses (20 poses x 6 protein conformations) of falvopiridol and methotrexate, we assigned amino acids semi-manually to binding **Site 1** (Q126, D127, D128, D128, V129, V129, V130, V130, M131, M131, G132, G132, T133, T133, L134, V178, V178, G179, G179, T180, T180, Q181, F182, F182, I183, I183, R191, R191, N387, N387, L388, G390, N391, N391, P392, Q393, Q393, A394, A394, A397, Q398, S443, A444, E446, L447, L447, F448, V449, V449, V450, V450, E451, K452, K453, K453, L454, I456, K473, D477), **Site 2** (L388, A394, A397, Q398, I399, V401, T402, L405, Q437, C438, S440, S441, V442, S443, A444, V445, E446, L447, F448, V450, K473, D477, L478, M481, R482, P485, S486, A517, A520, S521, A524), **Site 3** (A397, V401, F439, F439, S440, V442, V442, S443, S443, V445, V445, E446, E446, L447, V449, V533, V534, V534, S535, S535, V536, A537, T538, T538, L539, L540, M541, M541, T542, I543, F545) and **Site 4** (Y413, I423, Q424, Q424, N425, A427, A427, G428, G553, G553, L554, L554, L555, L555, V556, N557, N557, F578, F586, P602, C603, N604, Y605, A606, T607, L614, Q617, G618, I619, L621). **(A)** Colors red, blue, yellow, magenta, deep purple, orange, and green label Sites 1, 2, 3, and 4, overlap of Sites 1–2, 2–3, and 1–3. Although there is an overlap in the amino acids of sites, a docked pose can be assigned to a binding site based on the numbers of interacting amino acids of two sites with the small molecule. The overlap of binding sites indicates a pathway and a mechanism of transport by binding of a molecule somewhere to the pathway followed by its moving forward to a next site closer to the extracellular space. For example binding to Site 1, which seems to be the most accessible site, can be followed by the movement to Site 2. Site 2 includes R482, which position has an effect on substrate specificity, and may participate in substrate selection. Non-substrates may not penetrate deeply into this site to induce conformational changes necessary for moving forward on the pathway. Although the initial set of molecules (4 substrates and 2 non-substrates) provided a hint for binding site identification, their low number may not be sufficient for representative results. Therefore we investigated the docking of additional 21 substrates and 12 non-substrates. All poses ((25+14 molecules) x 20 poses x 6 protein conformations) were automatically assigned to binding sites. **(B)** Although a density plot of binding affinities indicates lower binding energies for substrates compared to non-substrates, there are also poses of non-substrates with low binding energies, thus *in silico* values cannot be used to characterize the binding of molecules. Therefore we determined the frequency of substrates and non-substrates in each binding site. **(C)** The number of substrates and non-substrates bound to each binding site was counted and normalized to the number of molecules in the two categories (substrate or not) and to the number of poses. The values are shown in the table. Binding Site 1 was highly populated and more non-substrate poses can be found in this site as compared to substrate poses. Although many poses of non-substrate molecules can be found in Site 2, still this is the site showing increased binding of substrates as compared to non-substrates. These results strongly indicate a distinguished role of Site 2 in substrate selection. When generating sophisticated *in silico* methods for predicting substrates of ABCG2, this Site 2 should be considered with higher weights in model building. Site 3, which is located in the hydrophobic transmembrane region between the two ABCG2 monomers, accommodates both substrates and non-substrates equally well. However, it is important to note that the access to this site might be limited *in vivo* (e.g. by the loop between the β1 and β2 strands of NBD), while *in silico* methods can place any fitting molecule into this volume.
(TIFF)

S9 Fig The R482G mutation has a pronounced effect on TM helix orientation and dynamics. In spite of the relatively short time periods we can cover in MD simulations, two interesting changes can be observed in this mutant, as compared to the wild type protein. In one of the simulations in chain B the distance between R482 (TH3) and Q398 (TH1) was largely increased in the mutant (top right panel). Moreover, in the other chain and in all of the simulations, the distance between R482 and Q398 increased faster, as compared to the wild type (top left panel). These observations strongly suggest that while these relative movements of TH1 and TH3 are observable in both the wild type and the mutant proteins, the interaction between TH1 and TH3 is more dynamic in the R482G variant as compared to the wild type. In addition, the distance between R482 and A517 significantly shortened in R482G (bottom panels), thus TH3 and TH4 can get into a more intimate contact, because of the lack of a long side chain in TH3. We could not observe significant differences in distances between TH3 and TH2 (middle panels) caused by this mutation.
(TIFF)

S10 Fig The CRAC mutant Y413S does not affect the dynamics of TH1. In order to characterize the relative orientation and movement of TH1, in which the mutated residue is located, the interaction of amino acids located at the two ends of TH1 were measured throughout the six trajectories for both the wild type and the Y413S mutant ABCG2. C_α_ distances of I399 (turquoise) and P485 (red) were calculated in addition to that of Y413 (blue) and V556 (magenta). We could observe a larger change only in one Y413S simulations, when the distance between these two residues were altered. However, similar changes could also be observed for a WT simulation. This most likely happens because the Leu-based motif resides in a loop region that may exhibit propensity for higher dynamics.
(TIFF)

S1 Table The *in silico* method (HMMTOP) [56,57] predicted better location of TM helices compared to experimentally concluded boundaries based on HA-insertion [54,55]. (TIF)

S2 Table Vina docking scores are better (lower values) for ABCG2 transported substrates, as compared to the values for non-substrates in most of the cases. For clarity only values for the initial set of 4 substrates and 2 non-substrates are listed.
(TIF)
