# Supplementary figures and images for: Jump into a New Fold—A Homology Based Model for the ABCG2/BCRP Multidrug Transporter
Source: PLoS One. 2016 Oct 14;11(10):e0164426. doi: 10.1371/journal.pone.0164426 (PMC5065228; doi:10.1371/journal.pone.0164426)

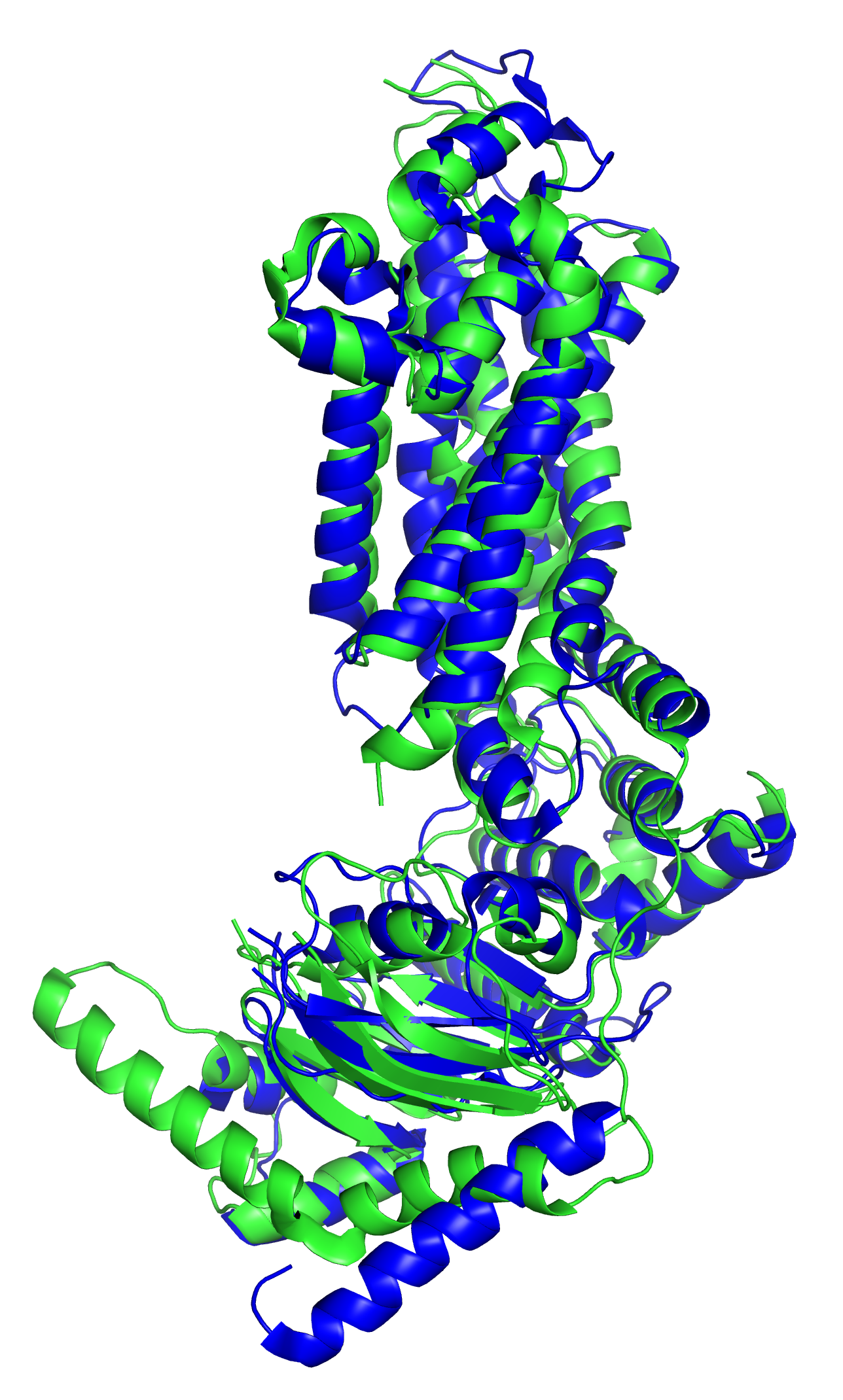

Supplement: S1 Fig — Chain A (ABCG5, green) and chain B (ABCG8, blue) from the heterodimer structure (PDBID: 5DO7) are highly similar (RMSD 2.1 Å) except in the linker region. (TIF) [file pone.0164426.s001.tif]

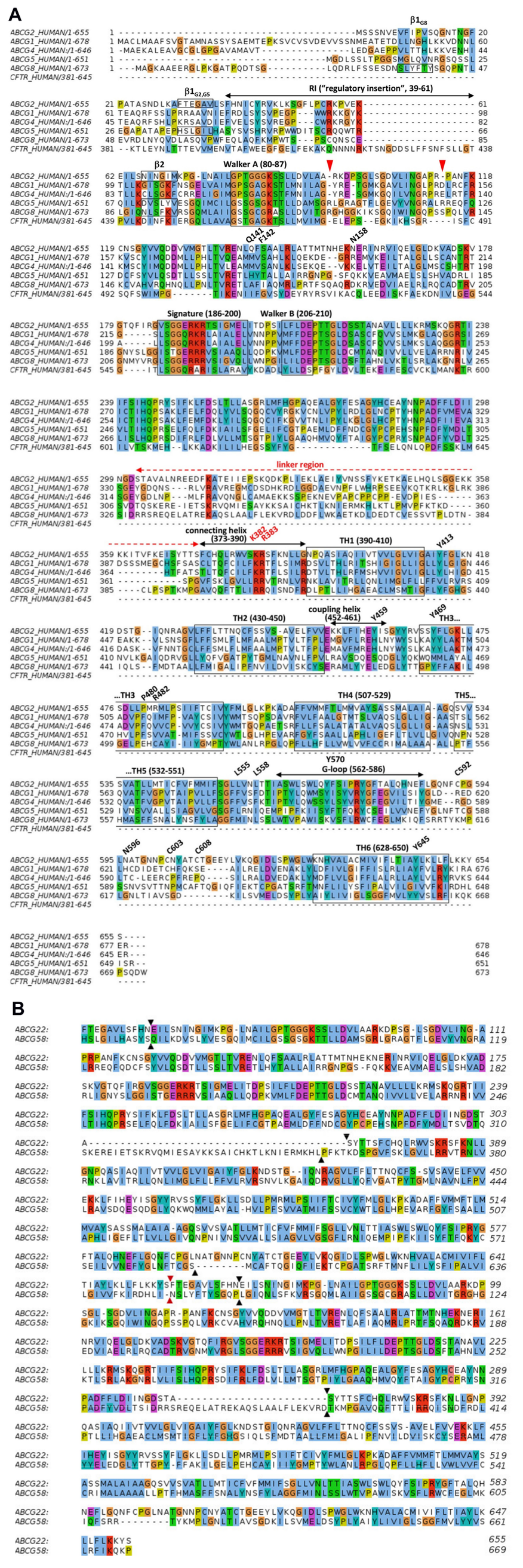

Supplement: S2 Fig — (A) Sequences of the ABCG proteins downloaded from the UniProt database (ABCG1_HUMAN, ABCG2_HUMAN, ABCG4_HUMAN, ABCG5_HUMAN, and ABCG8_HUMAN) were aligned employing ClustalW. ABCG2 N-terminus including NBD (a.a. 1–300) and CFTR NBD1 (CFTR_HUMAN, a.a. 381–645) were aligned separately, since CFTR NBD1 sequence has unique features, which result in suboptimal alignments even for demonstration purposes when aligned together with all ABCG proteins. The CFTR NBD1 was merged manually from this pairwise alignment into the multiple alignment. Since the CFTR transmembrane domain is a Type I exporter fold and not similar in either length or sequence to ABCG proteins, this TMD is not included in the alignment. Important regions and amino acids positions are labeled above the alignment and their numbering refers to ABCG2 positions. Since ABCG2 exhibit somewhat higher similarity to ABCG1 and ABCG4, the alignment of ABCG2 to ABCG5 and ABCG8 is not optimal at a few minor positions (labeled with red arrowheads). (B) We also generated pairwise alignments of the ABCG2 to the templates that was used for homology modeling and contains concatenated dimers of ABCG2 (labeled as ABCG22) and ABCG5-ABCG8 (labeled as ABCG58). The loop between the NBD β1 and β2 strands are highly different in length resulting in highly misaligned sequences of this region, thus the β1 sequence of ABCG8 was manually aligned to the β1 sequence of ABCG2. The linker region was not modeled because of low sequence similarities and its flexible missing parts in ABCG5-ABCG8. The ABCG2 sequence exhibits sufficiently high similarity to the templates (identity ~25% and similarity over 40%) that allows generating high quality homology models in the case of these ABC transmembrane proteins (Modeller tutorial and [34]). The alignment was drawn using Jalview, colored according to the ClustalX color scheme, and can be downloaded from http://abcg.hegelab.org. The monomer’s boundaries are indicated by red arrowheads, while locatio [file pone.0164426.s002.tif]

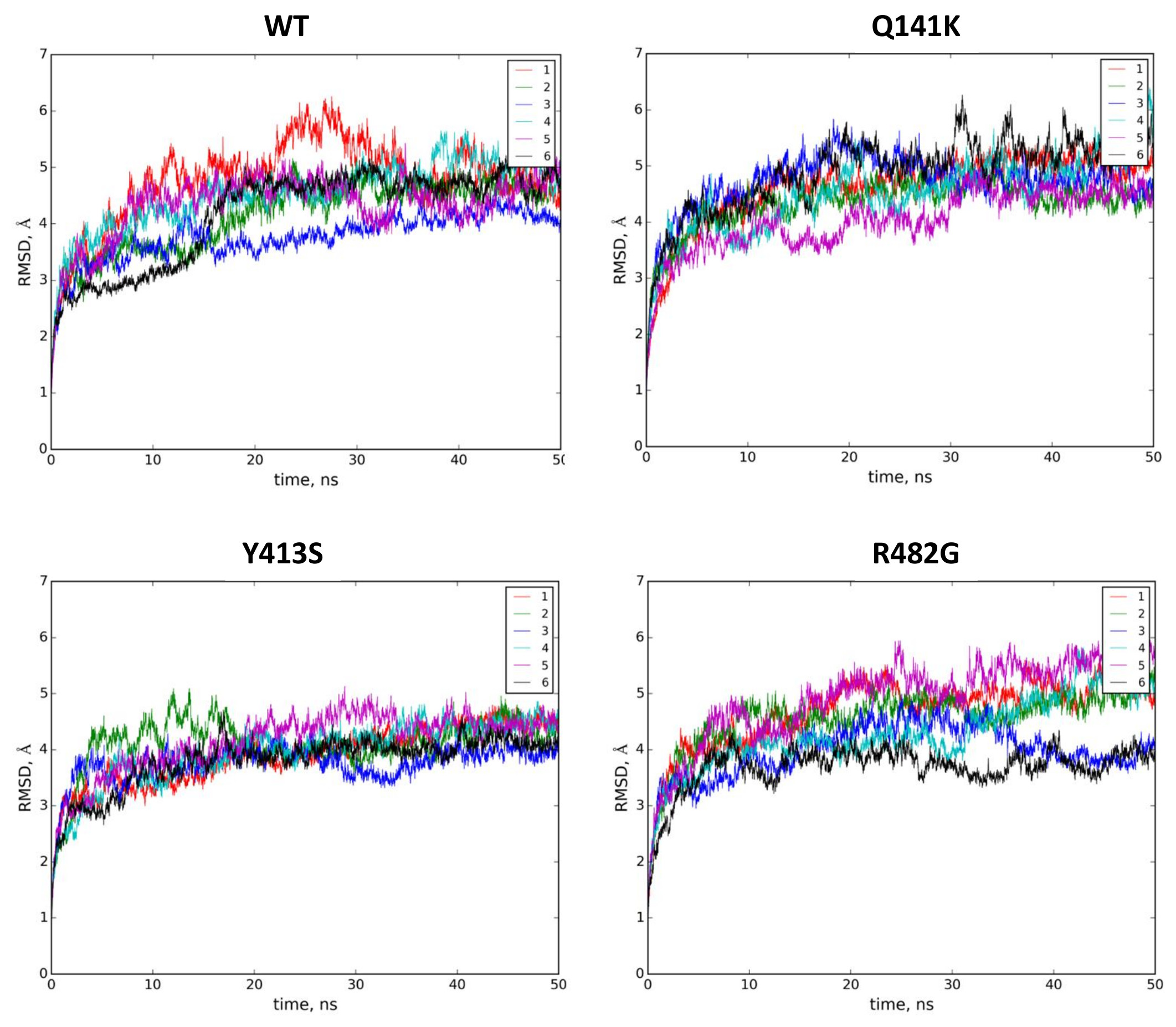

Supplement: S3 Fig — We performed six parallel 50 ns long MD simulations with every constructs embedded in a lipid bilayer. All of them exhibited a stable structure, with sufficiently stabilized RMSD and energy values that can be considered acceptable for such a large and stable system. Major distortions, which were published for MD simulations with crystal structures of other ABC transporters, could not be observed in the case of our structural model [38,39]. (TIFF) [file pone.0164426.s003.tiff]

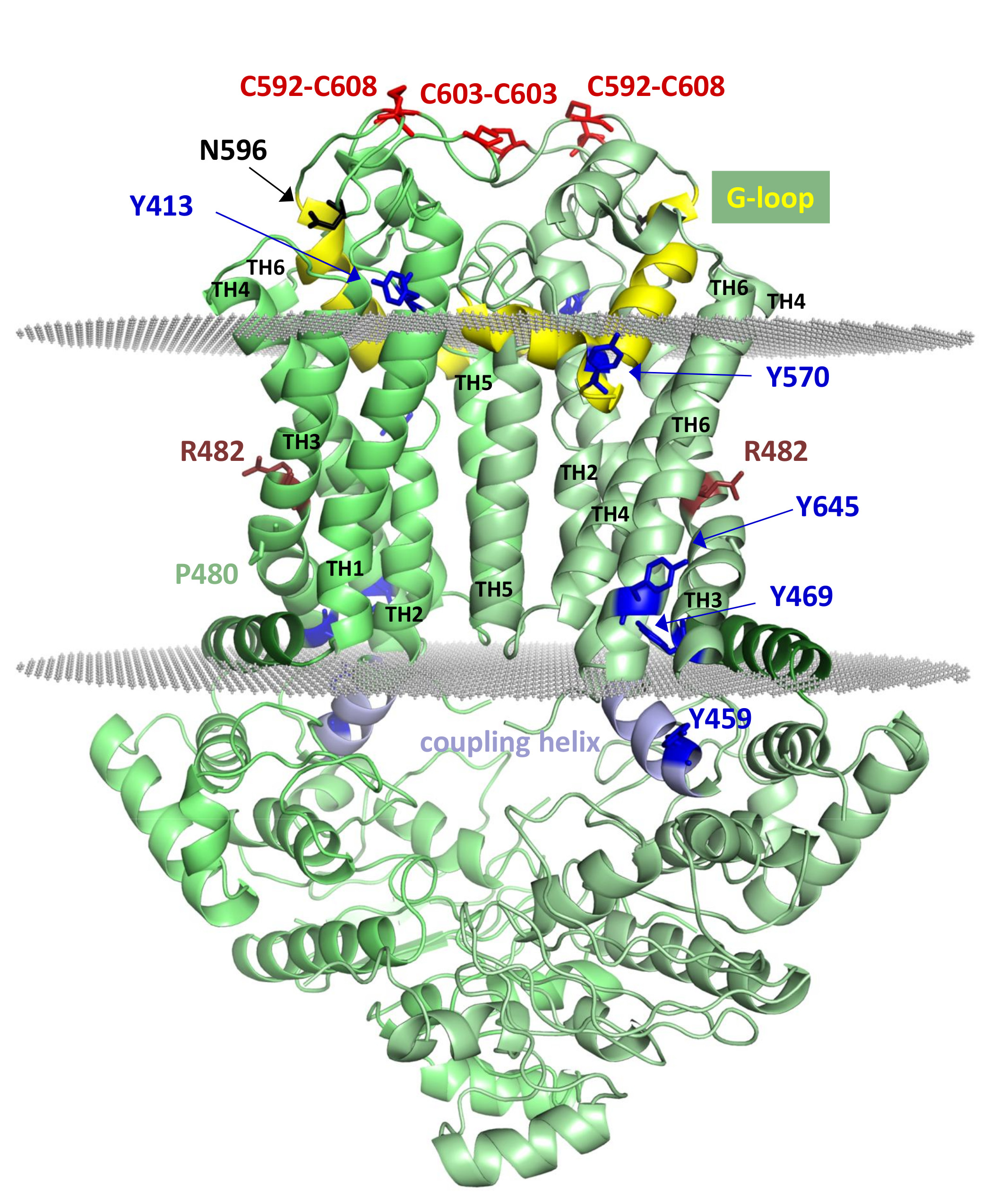

Supplement: S4 Fig — Cysteines (red) were constrained to form intramolecular (C592-C608) or intermolecular (C603-C603) disulfide bonds. The glycosylation site N596 (black) resides in a mobile loop indicated by molecular dynamics simulations. Amino acids between 562 and 586 form two consecutive helices, which are immersed partially into the bilayer and resembles to the P-loop of ion channels, thus we propose to name it as a G-loop (yellow). CRAC motives are labeled blue and the coupling helix light blue. The tyrosines in the potential CRAC motifs (Y413, Y459, Y469, Y570 and Y645, see text) are colored blue. R482 and P480, which proline creates a kink in the helix, are colored ruby and pale green, respectively. (TIFF) [file pone.0164426.s004.tiff]

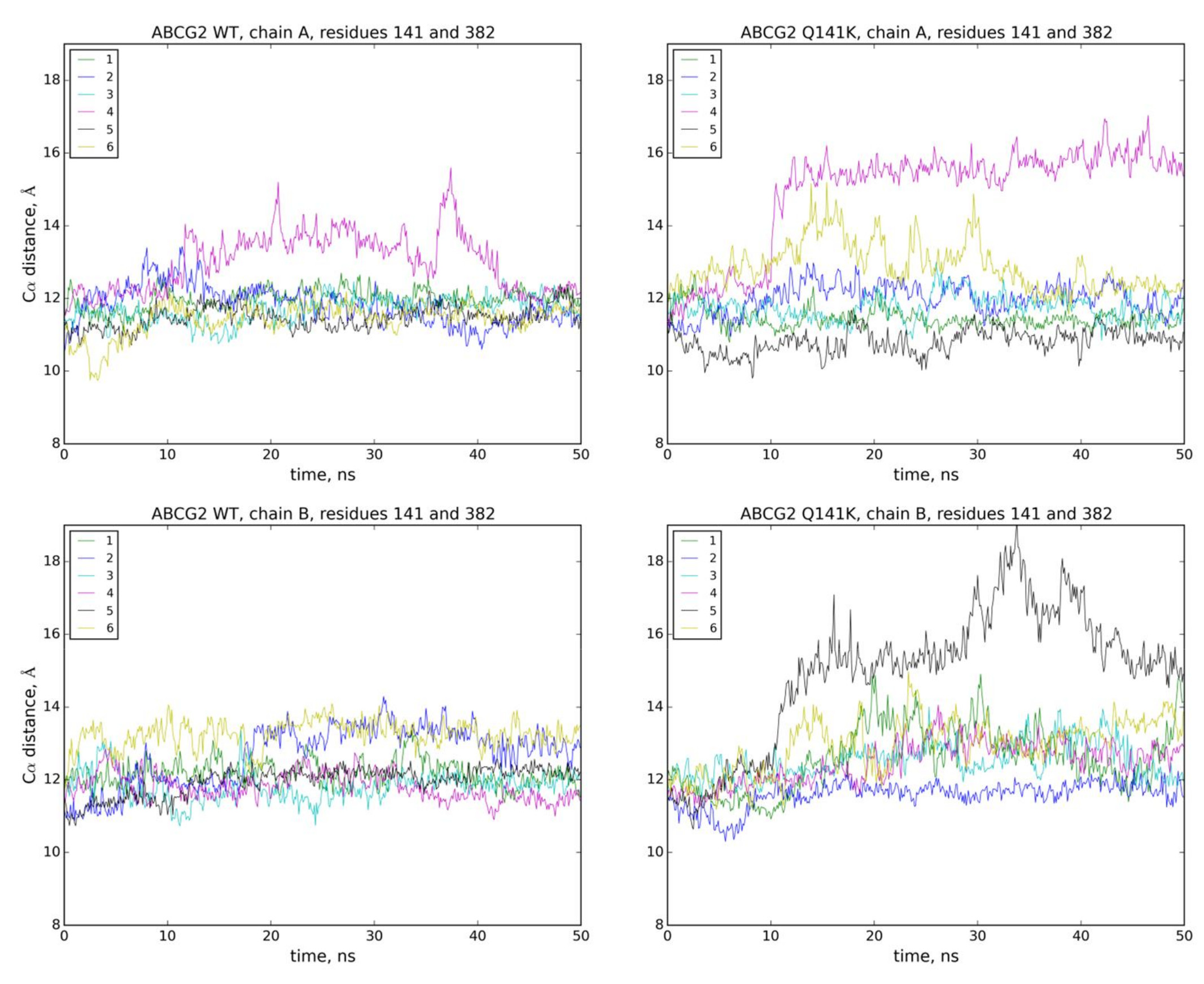

Supplement: S5 Fig — Although the Q141 side chain points towards N158 (Fig 2), the Q141K mutations does not exhibit a pronounced effect of the distances of the helices, in which these amino acids are located (not shown). However, the positively charged 141K interferes with the side chain of K382, which restrains F142 with R383 (see Fig 2). This is revealed by MD simulations showing increased distances and higher variability of distances between the Cα atoms of restudies 141 and 382 in the Q141K variant, as compared to the wild type protein. All the 5,000 frames were analyzed and every consecutive ten distance values were averaged to smooth the plots of all distance measurements in this study. (TIFF) [file pone.0164426.s005.tiff]

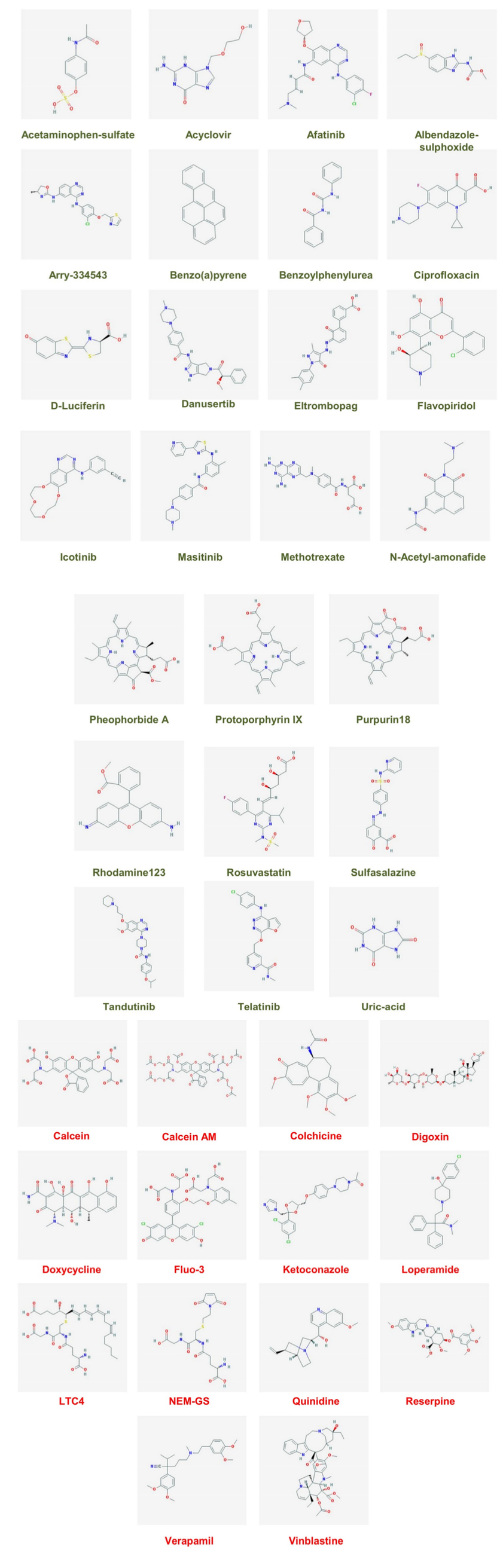

Supplement: S6 Fig — 3D and 2D structures were downloaded from PubChem and ChemSpider. ABCG2 substrates: Acetaminophen-sulfate (CID: 83939), Acyclovir (CID: 2022), Afatinib (CID: 10184653), Albendazole-sulphoxide (CID: 83969), Arry-334543 (CID: 42642648), Benzo(a)pyrene (CID: 2336), Benzoylphenylurea (CID: 74566), Ciprofloxacin (CID: 2764), D-Luciferin (CID: 5484207), Danusertib (CID: 11442891), Eltrombopag (CID: 9846180), Flavopiridol (CID: 5287969), Icotinib (CID: 22024915), Masitinib (CID: 10074640), Methotrexate (CID: 126941), N-Acetyl-amonafide (CID: 10064887), Pheophorbide A (CID: 5323510), Protoporphyrin IX (CID: 4971), Purpurin18 (CID: 5489047), Rhodamine123 (CID: 65218), Rosuvastatin (CID: 446157), Sulfasalazine (CID: 5359476), Tandutinib (CID: 3038522), Telatinib (CID: 9808844), and Uric-Acid (CID: 1175). Non-substrates: Calcein (CID: 65079), Calcein AM (ChemSpiderID: 346571), Colchicine (CID: 6167), Digoxin (ChemSpiderID: 206532), Doxycycline (CID: 54671203), Fluo-3 (ChemSpiderID: 94730), LTC4 (ChemSpiderID: 4444133), NEM-GS (CID: 443150), Ketoconazole (CID: 456201), Loperamide (CID: 3955), Quinidine (CID: 441074), Reserpine (CID: 5770), Verapamil (CID: 2520), and Vinblastine (ChemSpiderID: 12773). (TIF) [file pone.0164426.s006.tif]

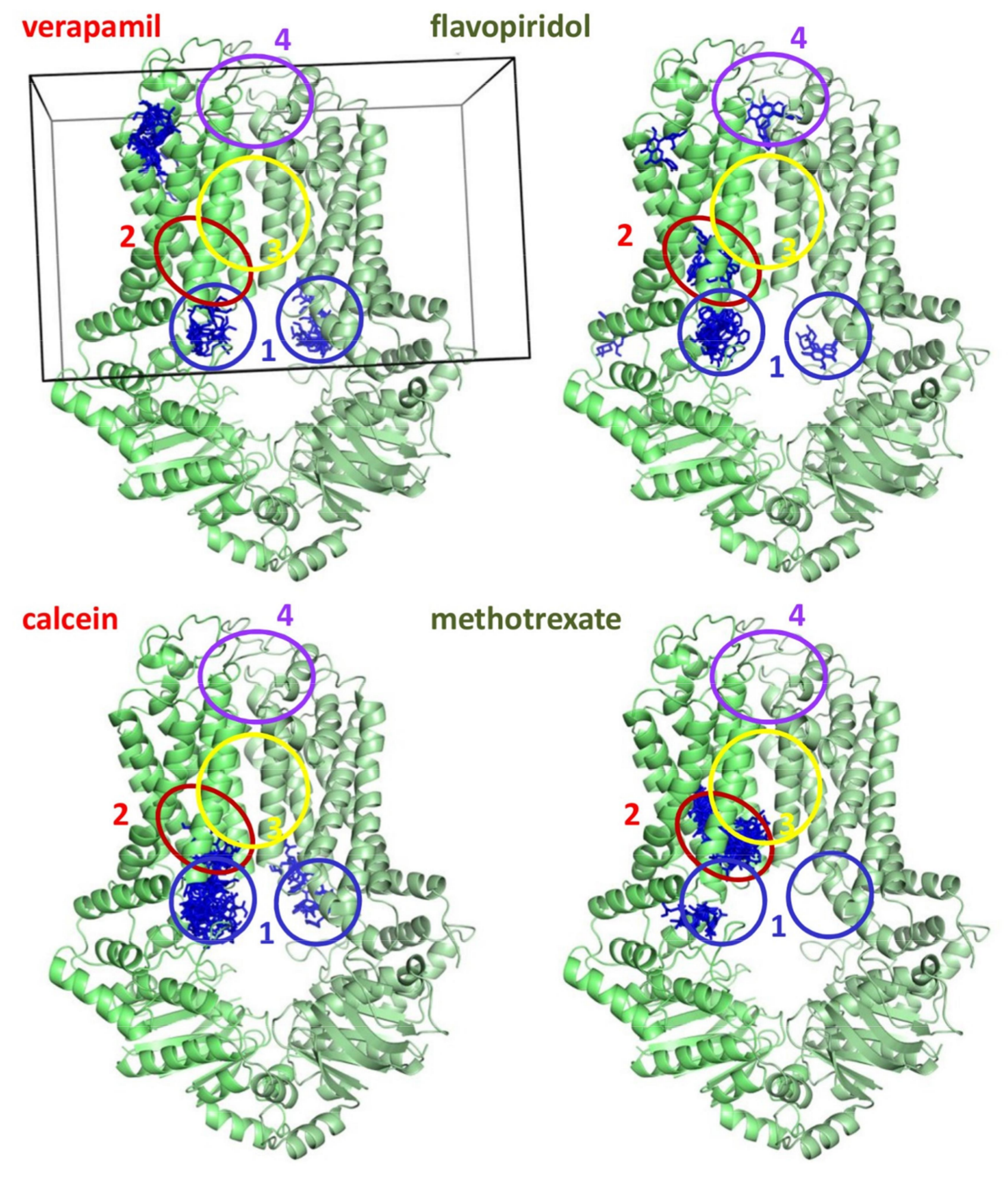

Supplement: S7 Fig — We performed in silico docking calculations for flavopiridol and methotrexate, two established ABCG2 substrates (green labels) as well as verapamil and calcein, which are not transported substrates of ABCG2 (red labels), to the six equilibrated conformation of the transporter. Although we could observe binding to Site 1 (blue) for all compounds, access of verapamil to Site 2 (red), which is the binding pocket including R482, was decreased compared to that of substrates. Calcein also exhibited a limited access to this site, without deep penetration into it. These observations suggest that these ABCG2 conformations can be employed in future studies for developing in silico methods to distinguish substrates and non-substrates. Moreover, Site 2 may be the gate that differentiates between toxic molecules and tolerated metabolites. The black box indicates the search space defined in all of our docking calculations. Docking to one of the six equilibrated conformations is shown. (TIFF) [file pone.0164426.s007.tiff]

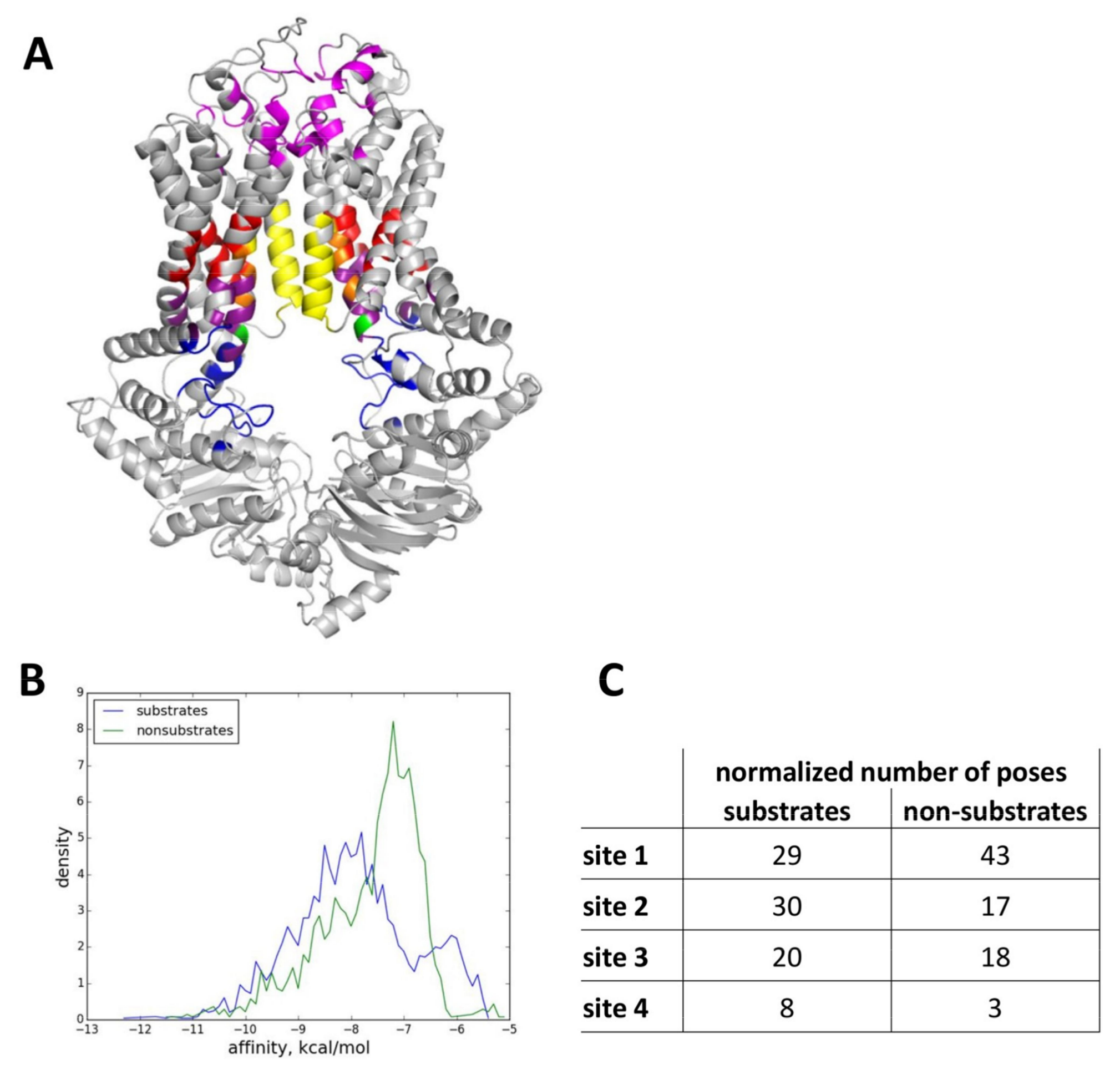

Supplement: S8 Fig — After visual inspection of all the binding poses (20 poses x 6 protein conformations) of falvopiridol and methotrexate, we assigned amino acids semi-manually to binding Site 1 (Q126, D127, D128, D128, V129, V129, V130, V130, M131, M131, G132, G132, T133, T133, L134, V178, V178, G179, G179, T180, T180, Q181, F182, F182, I183, I183, R191, R191, N387, N387, L388, G390, N391, N391, P392, Q393, Q393, A394, A394, A397, Q398, S443, A444, E446, L447, L447, F448, V449, V449, V450, V450, E451, K452, K453, K453, L454, I456, K473, D477), Site 2 (L388, A394, A397, Q398, I399, V401, T402, L405, Q437, C438, S440, S441, V442, S443, A444, V445, E446, L447, F448, V450, K473, D477, L478, M481, R482, P485, S486, A517, A520, S521, A524), Site 3 (A397, V401, F439, F439, S440, V442, V442, S443, S443, V445, V445, E446, E446, L447, V449, V533, V534, V534, S535, S535, V536, A537, T538, T538, L539, L540, M541, M541, T542, I543, F545) and Site 4 (Y413, I423, Q424, Q424, N425, A427, A427, G428, G553, G553, L554, L554, L555, L555, V556, N557, N557, F578, F586, P602, C603, N604, Y605, A606, T607, L614, Q617, G618, I619, L621). (A) Colors red, blue, yellow, magenta, deep purple, orange, and green label Sites 1, 2, 3, and 4, overlap of Sites 1–2, 2–3, and 1–3. Although there is an overlap in the amino acids of sites, a docked pose can be assigned to a binding site based on the numbers of interacting amino acids of two sites with the small molecule. The overlap of binding sites indicates a pathway and a mechanism of transport by binding of a molecule somewhere to the pathway followed by its moving forward to a next site closer to the extracellular space. For example binding to Site 1, which seems to be the most accessible site, can be followed by the movement to Site 2. Site 2 includes R482, which position has an effect on substrate specificity, and may participate in substrate selection. Non-substrates may not penetrate deeply into this site to induce conformational changes necessary for moving for [file pone.0164426.s008.tiff]

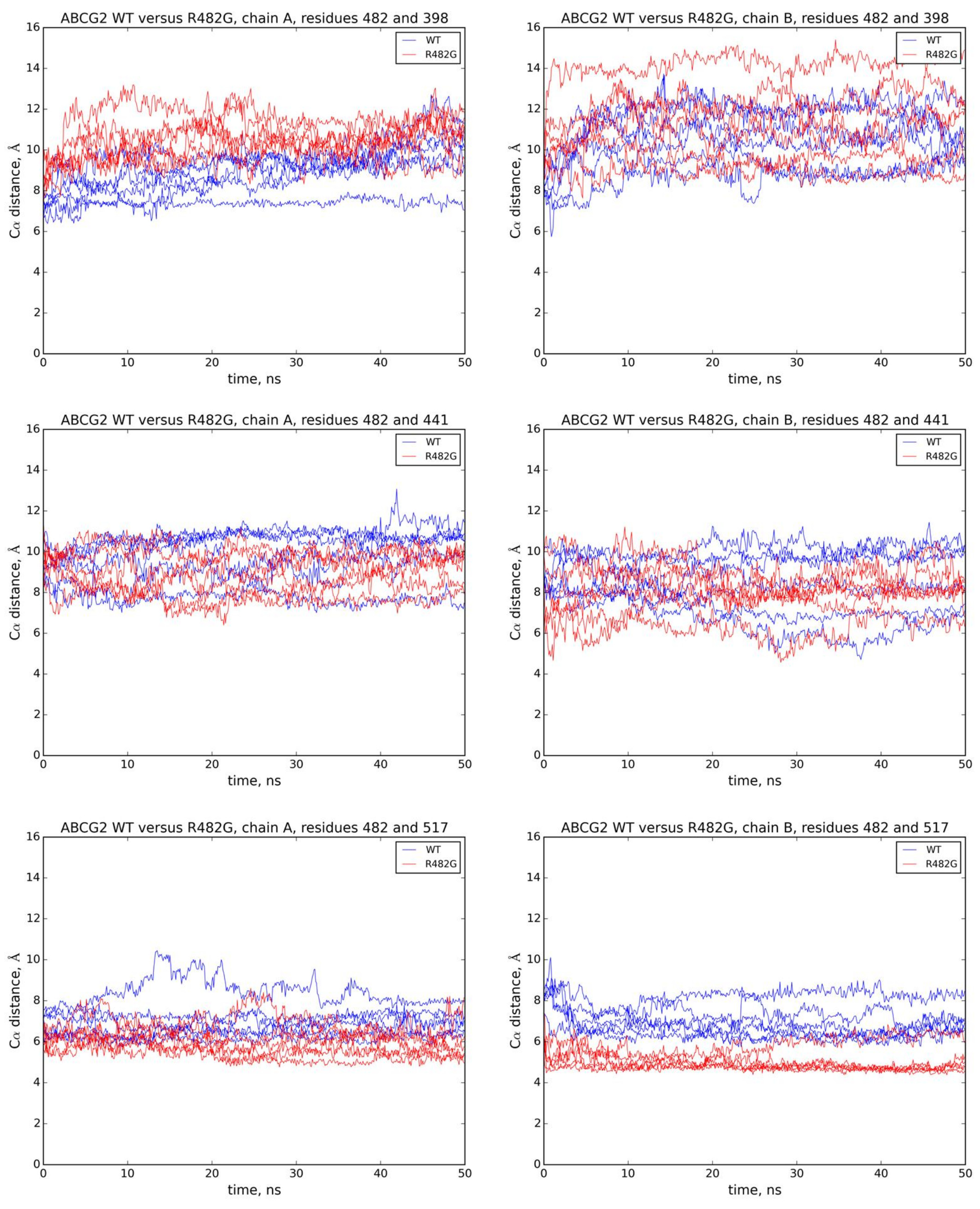

Supplement: S9 Fig — In spite of the relatively short time periods we can cover in MD simulations, two interesting changes can be observed in this mutant, as compared to the wild type protein. In one of the simulations in chain B the distance between R482 (TH3) and Q398 (TH1) was largely increased in the mutant (top right panel). Moreover, in the other chain and in all of the simulations, the distance between R482 and Q398 increased faster, as compared to the wild type (top left panel). These observations strongly suggest that while these relative movements of TH1 and TH3 are observable in both the wild type and the mutant proteins, the interaction between TH1 and TH3 is more dynamic in the R482G variant as compared to the wild type. In addition, the distance between R482 and A517 significantly shortened in R482G (bottom panels), thus TH3 and TH4 can get into a more intimate contact, because of the lack of a long side chain in TH3. We could not observe significant differences in distances between TH3 and TH2 (middle panels) caused by this mutation. (TIFF) [file pone.0164426.s009.tiff]

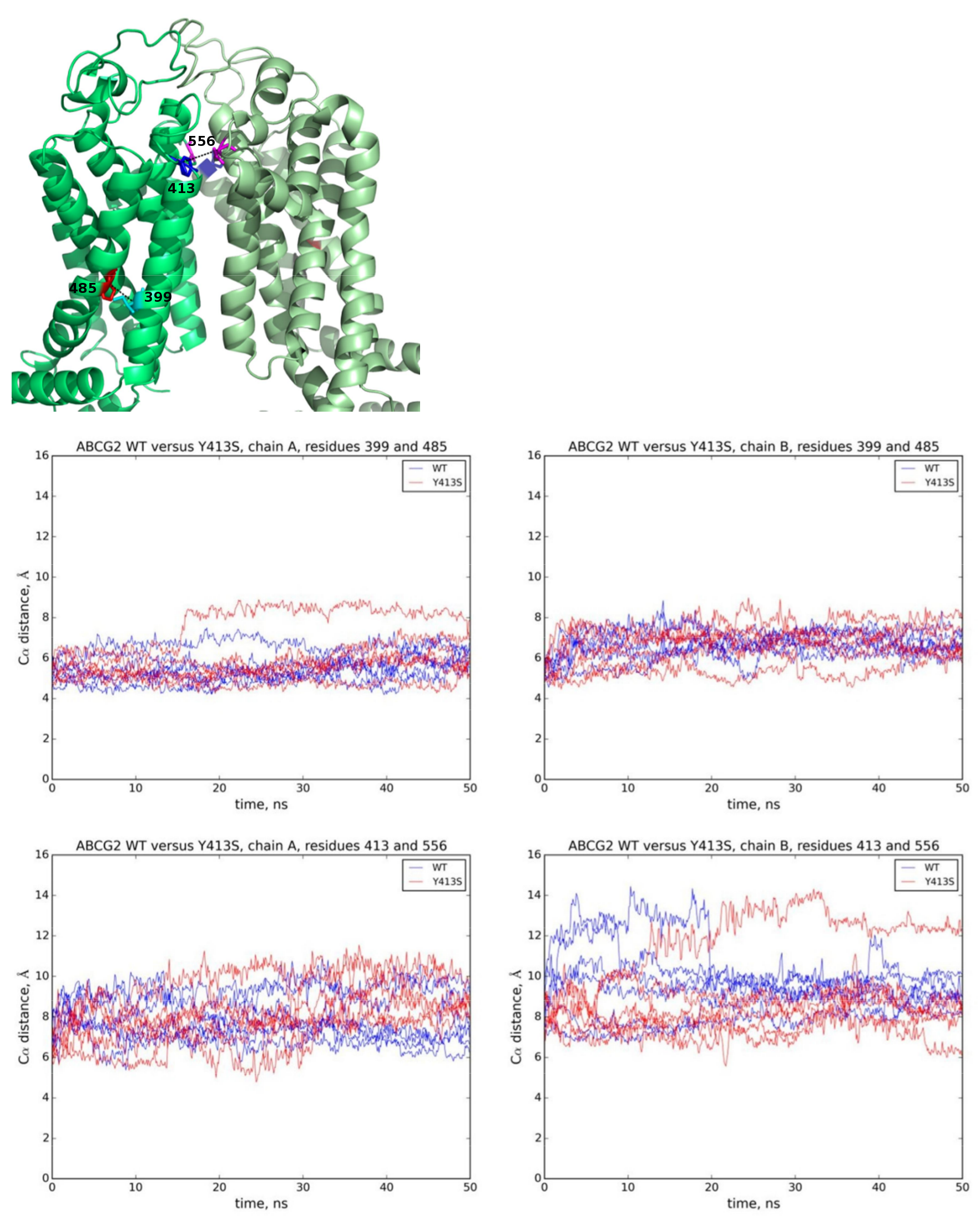

Supplement: S10 Fig — In order to characterize the relative orientation and movement of TH1, in which the mutated residue is located, the interaction of amino acids located at the two ends of TH1 were measured throughout the six trajectories for both the wild type and the Y413S mutant ABCG2. Cα distances of I399 (turquoise) and P485 (red) were calculated in addition to that of Y413 (blue) and V556 (magenta). We could observe a larger change only in one Y413S simulations, when the distance between these two residues were altered. However, similar changes could also be observed for a WT simulation. This most likely happens because the Leu-based motif resides in a loop region that may exhibit propensity for higher dynamics. (TIFF) [file pone.0164426.s010.tiff]

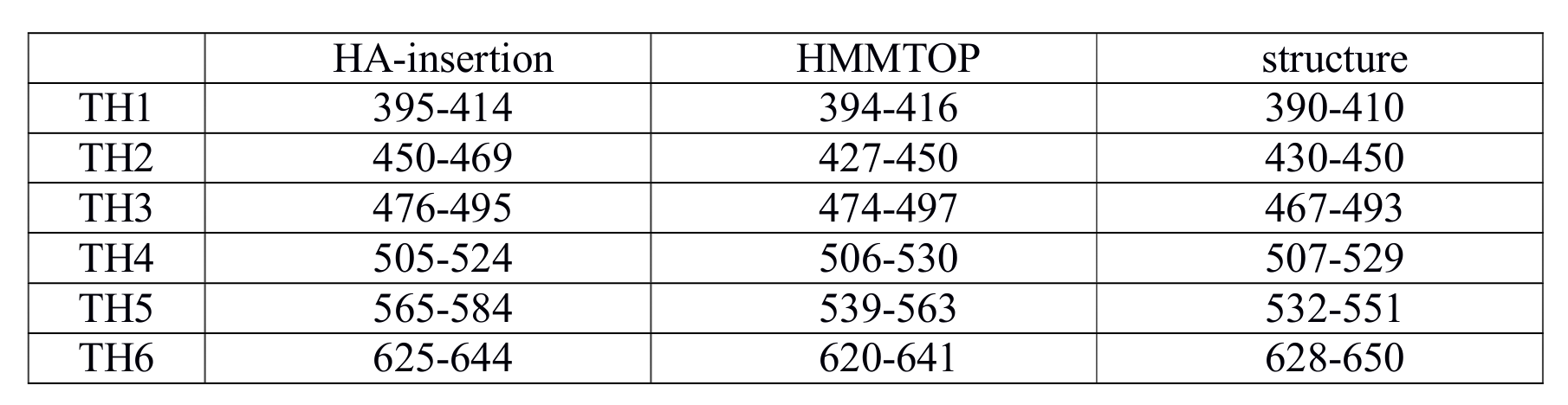

Supplement: S1 Table — (TIF) [file pone.0164426.s011.tif]

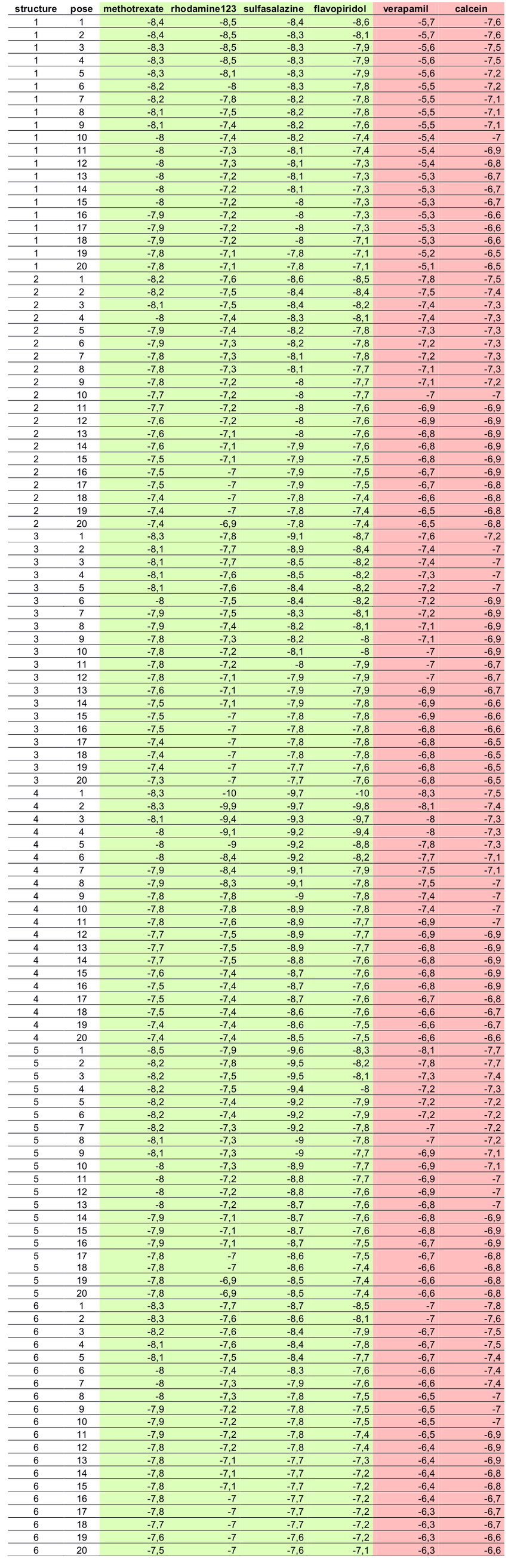

Supplement: S2 Table — For clarity only values for the initial set of 4 substrates and 2 non-substrates are listed. (TIF) [file pone.0164426.s012.tif]
